# What’s the difference? A gender perspective on understanding educational inequalities in all-cause and cause-specific mortality

**DOI:** 10.1186/s12889-018-5940-5

**Published:** 2018-09-10

**Authors:** Karen van Hedel, Frank J. van Lenthe, Joost Oude Groeniger, Johan P. Mackenbach

**Affiliations:** 1 Department of Public Health, Erasmus MC, P.O. Box 2040, 3000 CA Rotterdam, The Netherlands; 2 Max Planck Institute for Demographic Research, Rostock, Germany

**Keywords:** Education, Gender differences, Socioeconomic inequalities, Mortality

## Abstract

**Background:**

Material and behavioural factors play an important role in explaining educational inequalities in mortality, but gender differences in these contributions have received little attention thus far. We examined the contribution of a range of possible mediators to relative educational inequalities in mortality for men and women separately.

**Methods:**

Baseline data (1991) of men and women aged 25 to 74 years participating in the prospective Dutch GLOBE study were linked to almost 23 years of mortality follow-up from Dutch registry data (6099 men and 6935 women). Cox proportional hazard models were used to calculate hazard ratios with 95% confidence intervals, and to investigate the contribution of material (financial difficulties, housing tenure, health insurance), employment-related (type of employment, occupational class of the breadwinner), behavioural (alcohol consumption, smoking, leisure and sports physical activity, body mass index) and family-related factors (marital status, living arrangement, number of children) to educational inequalities in all-cause and cause-specific mortality, i.e. mortality from cancer, cardiovascular disease, other diseases and external causes.

**Results:**

Educational gradients in mortality were found for both men and women. All factors together explained 62% of educational inequalities in mortality for lowest educated men, and 71% for lowest educated women. Yet, type of employment contributed substantially more to the explanation of educational inequalities in all-cause mortality for men (29%) than for women (− 7%), whereas the breadwinner’s occupational class contributed more for women (41%) than for men (7%). Material factors and employment-related factors contributed more to inequalities in mortality from cardiovascular disease for men than for women, but they explained more of the inequalities in cancer mortality for women than for men.

**Conclusions:**

Gender differences in the contribution of employment-related factors to the explanation of educational inequalities in all-cause mortality were found, but not of material, behavioural or family-related factors. A full understanding of educational inequalities in mortality benefits from a gender perspective, particularly when considering employment-related factors.

**Electronic supplementary material:**

The online version of this article (10.1186/s12889-018-5940-5) contains supplementary material, which is available to authorized users.

## Background

Higher levels of education are related to lower rates of all-cause and cause-specific mortality in most European countries including the Netherlands [1–6]. Prior studies highlighted the importance of material factors (e.g., income, type of health insurance, and financial difficulties) and behavioural factors (e.g., smoking, excessive alcohol consumption, and diet) in explaining educational inequalities in mortality [7–12]. Educational gradients in mortality have been found for both men and women [4, 5]. Absolute mortality differences by education are generally larger for men than for women, but gender differences in relative mortality differences by education are less clear [13, 14]. These findings suggest that explanations for the educational gradient may also differ for men and women, an issue that hardly received attention thus far.

Indeed, two mechanisms may explain why material and behavioural factors contribute differently to the explanation of educational inequalities in mortality between men and women. Firstly, the impact of education on material, employment-related and behavioural factors may differ. For example, socioeconomic inequalities in overweight are smaller for Dutch men than for Dutch women [15], and educational inequalities in smoking prevalence were larger for men than for women in the European Union [16]. Secondly, the effect of material, employment-related and behavioural factors on mortality may differ. For example, unemployment is more strongly related to mortality for men than for women, which may be the result of employment status being more central to men’s identities than to women’s [17].

In addition, family-related factors may play a role in generating gender differences in educational inequalities in mortality. The educational gradient in family factors may be different for men and women. Indeed, higher educated men and women are more likely to ever get married than their lower educated counterparts, but this marriage gap seems to be larger for men than for women [18, 19]. Additionally, higher educated Dutch women are more likely to remain childless than low educated women, whereas the proportion of Dutch men that remains childless is similar across different educational levels [19]. Additionally, mortality differentials by marital status, living arrangement and parenthood status have been found; mortality is lower for married individuals, individuals living with a partner, or parents, than for their unmarried, living alone, and childless counterparts, respectively [20–22]. These family factors may also differentially impact mortality of men and women. For example, marriage is more protective of health and mortality for men than for women [23]. There seem to be no clear gender differences in the association between parenthood and mortality [24, 25]. To our knowledge the contribution of these factors to educational inequalities in mortality has not yet been studied.

A proper understanding of the underlying causes of inequalities in mortality is needed for adequate interventions and policies aimed at bridging the health gap between the higher and lower educated. Perhaps surprisingly, and despite good reasons to assume that the explanation of socioeconomic inequalities in health may differ by gender, only few studies investigated this with a specific gender perspective [26–29]. The aim of this article was to examine whether explanations for relative educational inequalities in mortality differed between men and women. We examined multiple material, employment-related, behavioural and family-related factors, using data from a Dutch cohort study linked on an individual level to registry data with almost 23 years of mortality follow-up.

## Methods

Data came from the prospective GLOBE study (the Dutch acronym for Health and Living Conditions of the Population of Eindhoven and surroundings) [30] initiated to quantitatively assess mechanisms and factors explaining socioeconomic inequalities in health in the Netherlands [31]. Baseline information was collected through a postal survey in 1991. This survey was distributed among 27,070 non-institutionalized respondents aged 15 to 74 years living in Eindhoven, a city in the South of the Netherlands, and its surrounding municipalities [32]. The response for this postal survey was 70.1%, leaving 18,973 respondents in the baseline sample. This sample was then on an individual level linked (94%) to almost 23 years of mortality follow-up from Statistics Netherlands.

Men and women aged 25 to 74 years were included in our study (*n* = 15,534). We excluded those who reported at least one of six severe chronic diseases (chronic obstructive pulmonary disease, heart disease, stroke, renal disease, diabetes, or cancer) at baseline (*n* = 2500). Having a chronic illness may influence an individual’s survival, but it may also affect their explanatory factors such as health behaviours. For example, individuals may improve their health behaviours after a health scare due to chronic illness, e.g. cease smoking, eat healthier or become more physically active. However, our explanatory variables may also have influenced the likelihood of becoming chronically ill, as unhealthy behaviours increase the chance of becoming chronically ill [33]. As no information was available prior to our baseline data in 1991, we cannot disentangle how these two mechanisms might be working together due to lack of a time dimension. Overall, our analyses included 6099 men and 6935 women.

### Levels of education

Educational level was used to represent the socioeconomic position of the individual, as it is commonly used as an indicator for socioeconomic status in the Netherlands. We distinguished 4 levels of educational attainment with the following equivalent levels of the International Standard Classification of Education (ISCED) [34]: primary education only (“lowest”, ISCED 0 and 1), lower vocational school and lower secondary school (“low”, ISCED 2), intermediate vocational school and intermediate or higher secondary school (“mid”, ISCED 3 and 4), and higher vocational school and university (“high”, ISCED 5 and 6).

### Explanatory variables: material, employment-related, behavioural and family-related factors

All explanatory factors were derived from the postal survey collected in 1991. Financial problems, housing tenure and health insurance were included as material factors. Financial problems were measured by asking the respondents if they had any difficulties paying bills, food, rent, electricity, etcetera during the previous year (no difficulties, some difficulties, and big difficulties). With regards to housing tenure, we distinguished between individuals owning and those renting their home. The two possible types of health insurance were public and private insurance. As employment-related factors, we included the respondent’s employment status (employed; unemployed; retired; others, e.g. students or homemakers) and the occupation of the main breadwinner (professional; white-collar; blue-collar occupations; not in the workforce) [35].

The behavioural factors included in this study were alcohol consumption, smoking, physical activity in leisure time, physical activity in sports, and body mass index (BMI). Alcohol consumption (weekly number of drinks) was calculated from information on the number of days per week the respondent drank alcoholic drinks and the number of alcoholic drinks (units) consumed on such a day; no consumption, light consumption (1 to 14 drinks for men, 1 to 7 drinks for women), moderate consumption (15 to 21 drinks for men, 8 to 14 drinks for women), and heavy consumption (22 or more drinks for men, 15 or more drinks for women; same cut-off as used by Statistics Netherlands to define excess alcohol consumption). Regarding smoking status, we distinguished current smokers from former smokers and never smokers. Physical activity in leisure time was measured by two questions “How many hours of your leisure time do you spend in total per week on working in the garden, biking, walking, walking the dog?” and “How many hours of your leisure time do you spend in total per week on chores, fixing the house, repairs?”. Sports physical activity was measured by the question “Do you exercise?”. Both physical activity questions had the following 4 answer categories; (i) no, (almost) never (“inactive”), (ii) yes, less than 1 h a week (“little active”), (iii) yes, approximately 1 to 2 h a week (“moderately active”), and (iv) yes, 2 h a week or more (“active”). BMI (kg/m^2^) was calculated from self-reported weight and height (underweight, BMI < 20; normal weight, 20≤BMI < 25; overweight, 25≤BMI≤30; or obese, BMI > 30). BMI was included here as it is mainly determined by behaviour.

As family-related factors we included marital status, living arrangement and number of children. Marital status was categorized into currently married, previously married (i.e., divorced and widowed) and never married. We also included living arrangement (living together with a partner or living alone). Lastly, we distinguished between no, one, two, and three or more children.

### Outcome measures

Mortality data were obtained from Statistics Netherlands. The GLOBE baseline survey (April 1, 1991) was linked to death registry data until December 31, 2013, allowing for almost 23 years of mortality follow-up. We examined educational inequalities in all-cause mortality, as well as in 4 categories of causes of death: mortality from cancer, cardiovascular disease (CVD), other diseases, and external causes. The International Classification of Disease, 10th revision codes [36] for the causes of death included in each of these categories are provided in the footnote of Table 4.

### Analysis

Missing values for the explanatory factors, but not educational attainment or mortality, were handled by applying multiple imputations (M = 5) [37, 38]. We imputed missing values based on all other factors included in the analysis.

Our analytical strategy consisted of four steps, and essentially follows the steps of a mediation analysis [39]. We thus assume causal effects of education on mortality, of education on the mediators and of the mediators on mortality, as would be done within a mediation analysis. However, we understand that our observational study cannot lead to causal effects, and therefore we refer to our results as associations. First, we calculated hazard ratios (HR) and their 95% confidence intervals (CI) for the association between educational level and mortality for men and women, using Cox proportional hazard models with age as the time scale (also referred to as model 0). Second, age-standardised prevalence rates of the explanatory factors by educational level were calculated for men and women. Third, Cox proportional hazard models were used to assess the association between each explanatory factor and mortality, adjusted for education (added as an independent variable to the models). Fourth, we estimated hazard ratios for education after inclusion of the material, employment-related, behavioural and/or family-related factors in Cox proportional hazard models. The contributions of these factors to educational inequalities in mortality were then estimated based on the changes in the hazard ratios for education after inclusion of these explanatory factors (adjusted models). The absolute change in the hazard ratios (HR) for education after the explanatory factors were included, was calculated by HR_model 0_ – HR_adjusted_. The relative change was calculated as follows: (HR_model 0_ – HR_adjusted_)/(HR_model 0_–1). We estimated the contributions of each explanatory factor to educational inequalities in mortality separately and for the broader categories of factors. Confidence intervals of the contributions to educational inequalities in mortality were calculated using a bootstrap with 5000 repetitions; 1000 repetitions per imputed dataset. The same procedure was used to assess the contributions of the explanatory factors to educational inequalities in cause-specific mortality. Cause-specific mortality was analysed within a competing risks framework [40]; as we were interested in mortality from a specific cause, and wanted to account for the fact that individuals may die from other causes than the one we were interested in. Gender differences in the contribution of material, employment, behavioural and family factors to the explanation of educational inequalities in all-cause and cause-specific mortality were assessed by comparing the estimated 95% confidence intervals of men and women. Gender differences were determined based on non-overlapping confidence intervals of the contribution of factors to educational inequalities in mortality for men and women.

The Cox proportional hazard models and the multiple imputation strategy were performed using Stata SE version 14.1. The bootstrapped confidence intervals were calculated in R version 3.3.1.

## Results

**Fig. 1:**
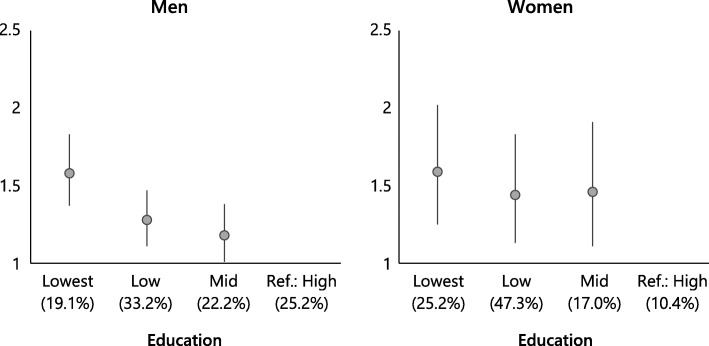
Mortality hazard ratios by education for men and women. Ref.: Reference category. The analysis was controlled for age. Proportion of men and women aged 25 to 74 years in each educational category are shown in brackets

As compared to those with the highest levels of education, significantly increased hazard ratios were found for those with lower levels of education (Fig. 1). Whereas an inverse educational gradient in mortality was found for men, reasonably similar hazard ratios were found at all three lower levels of education for women. Proportions of men and women in each educational category, with their 95% confidence intervals, are presented in Additional file 1: Table S1.

### Distribution of explanatory factors by educational level

Inverse educational gradients were found for all material and employment-related factors, but noticeable differences were found in the size of these gradients between men and women (Table 1). The educational gradient was larger for men than women with regards to the proportion privately insured, unemployed and blue-collar occupation of the breadwinner.

**Table 1:** Educational gradients in explanatory factors for men and women

	Men				Women				Testing for a gender difference in gradient (*p* value) ^a^
	Lowest	Low	Mid	High	Lowest	Low	Mid	High	
Material factors									
Financial difficulties									
No	65.1%	76.2%	83.0%	92.6%	62.9%	78.8%	83.6%	90.0%	0.325
Some	27.4%	21.3%	14.8%	6.5%	29.5%	17.7%	13.8%	9.0%	0.430
Big	7.6%	2.6%	2.1%	0.9%	7.6%	3.5%	2.7%	1.0%	0.303
Housing tenure									
Owned home	29.5%	48.4%	62.0%	76.5%	34.2%	53.0%	67.3%	74.2%	0.112
Rented home	70.5%	51.6%	38.0%	23.5%	65.8%	47.0%	32.7%	25.8%	"
Health insurance									
Private	7.8%	27.0%	50.6%	79.0%	15.9%	31.2%	48.3%	66.6%	< 0.001
Public	92.2%	73.0%	49.4%	21.0%	84.1%	68.8%	51.7%	33.4%	"
Employment-related factors									
Employment									
Employed	46.7%	60.2%	61.0%	66.1%	18.9%	24.4%	34.0%	41.0%	0.932
Unemployed	27.6%	12.7%	9.6%	4.6%	9.7%	6.4%	4.9%	7.1%	< 0.001
Retired	25.1%	26.3%	27.9%	28.4%	6.9%	7.2%	10.0%	15.8%	0.065
Other	0.5%	0.8%	1.6%	1.0%	64.4%	62.0%	51.1%	36.1%	< 0.001
Occupation of the breadwinner									
Professional	4.2%	16.8%	44.0%	85.3%	10.9%	25.5%	49.6%	77.4%	< 0.001
White collar	15.0%	23.7%	26.9%	8.5%	14.2%	22.3%	22.2%	11.7%	0.001
Blue collar	78.4%	57.9%	27.0%	4.9%	52.4%	38.1%	18.9%	5.6%	< 0.001
Not in the workforce	2.5%	1.6%	2.2%	1.3%	22.6%	14.2%	9.2%	5.3%	0.001
Behavioural factors									
Alcohol consumption									
No	20.0%	13.0%	10.9%	6.7%	44.9%	31.5%	21.2%	17.2%	0.005
Light	54.9%	61.2%	62.7%	67.6%	38.5%	47.8%	48.6%	51.7%	0.618
Moderate	8.5%	11.4%	12.8%	15.1%	11.5%	13.5%	18.6%	20.9%	0.147
Heavy	16.7%	14.4%	13.7%	10.5%	5.1%	7.3%	11.5%	10.2%	< 0.001
Body mass index (BMI)									
Underweight	4.3%	3.4%	3.4%	4.0%	7.2%	8.5%	10.1%	12.1%	0.071
Normal weight	46.2%	46.9%	55.1%	61.8%	49.1%	56.2%	62.1%	65.0%	0.844
Overweight	41.8%	44.6%	37.5%	32.4%	32.3%	28.5%	23.5%	17.7%	0.118
Obese	7.6%	5.1%	4.0%	1.8%	11.4%	6.7%	4.3%	5.2%	0.989
Smoking									
Current	54.8%	45.2%	38.2%	35.4%	42.1%	32.7%	28.9%	19.9%	0.009
Former	34.1%	40.4%	43.5%	42.9%	21.7%	28.2%	30.7%	32.5%	0.181
Never	11.1%	14.5%	18.3%	21.7%	36.2%	39.1%	40.4%	47.7%	0.056
Leisure activity									
Inactive	15.5%	11.9%	12.2%	10.1%	18.6%	11.7%	10.8%	7.2%	0.003
Little	12.9%	14.0%	14.4%	16.3%	16.8%	16.6%	15.3%	18.4%	0.119
Moderate	22.5%	25.1%	26.0%	28.1%	29.7%	27.5%	29.9%	26.7%	0.005
Active	49.1%	49.0%	47.5%	45.5%	34.9%	44.3%	44.0%	47.7%	< 0.001
Sports activity									
Inactive	73.3%	60.9%	51.6%	44.0%	72.9%	56.9%	46.2%	43.1%	0.320
Little	4.8%	6.6%	9.4%	8.4%	7.2%	8.0%	8.7%	11.3%	0.391
Moderate	9.9%	14.3%	16.6%	23.8%	12.4%	21.5%	26.9%	23.3%	0.302
Active	12.0%	18.2%	22.4%	23.8%	7.4%	13.6%	18.2%	22.3%	0.004
Family-related factors									
Marital status									
Currently married	73.6%	82.1%	81.1%	77.7%	71.5%	78.0%	72.0%	65.9%	0.001
Previously married	10.2%	7.5%	7.5%	8.7%	20.4%	15.4%	14.3%	11.2%	0.025
Never married	16.2%	10.4%	11.4%	13.6%	8.2%	6.6%	13.7%	22.9%	< 0.001
Living arrangement									
Living with partner	78.0%	86.9%	86.4%	84.2%	75.7%	81.5%	76.7%	73.0%	0.001
Living alone	22.0%	13.1%	13.6%	15.8%	24.3%	18.5%	23.3%	27.0%	"
Number of children									
0	26.0%	18.8%	22.3%	22.0%	12.2%	15.2%	21.8%	32.1%	< 0.001
1	16.6%	14.0%	12.2%	7.3%	14.4%	12.7%	10.8%	6.9%	0.275
2	32.7%	38.5%	37.5%	35.4%	42.3%	38.5%	31.5%	29.0%	< 0.001
3 or more	24.6%	28.7%	28.1%	35.3%	31.0%	33.6%	35.9%	32.0%	0.015

*Notes*. Age-standardised towards the age distribution of men and women observed in the data. The imputed values resulting from our multiple imputations strategy were also included in these distributions. ^a^ The *p* value of the difference in the educational gradient for men and women came from interaction models (education × gender) in which we additionally controlled for age and age × gender interactions

Educational gradients were also found for the behavioural factors, with sometimes contrasting directions between men and women. Specifically, the educational gradient for not consuming any alcohol was weaker for men than for women. Whereas heavy alcohol consumption decreased with increasing educational levels for men, it increased with education for women. The observed gradient in current smoking by educational level was smaller for men than for women, but in the same direction. The proportion of men being moderately active in leisure activities increased with higher levels of education, but the proportion of women being moderately active was similar across educational levels. The educational gradient in being active in leisure activities was similar across educational levels for men, but increased with increasing educational levels for women. The observed educational gradient in being active in sports activity was in the same direction for men and women, although smaller for men than for women.

Educational gradients were least clear for the family-related factors. The proportion of currently married persons was lowest among low educated men and among high educated women. Living alone was less common for men with higher education than those with lower education, but for women it was slightly higher for those with higher levels of education. With increasing levels of education, childlessness slightly decreased for men, but increased for women.

### Explanatory factors and their association with mortality

The associations of all material factors with mortality had comparable magnitudes for both men and women (Table 2). For the employment-related factors, some gender differences were found. Unemployment was associated with higher mortality for men (hazard ratio (HR) = 1.84, 95% confidence interval (CI): 1.58, 2.14), but not for women (HR = 1.26, 95% CI: 0.90, 1.64). In contrast, blue-collar occupation of the breadwinner was not associated with higher mortality for men (HR = 1.03, 95% CI: 0.89, 1.19), but it was associated with higher mortality for women (HR = 1.31, 95% CI: 1.13, 1.52). Being a former smoker, being previously married, and living alone was more strongly associated with mortality for men (former smoker vs. never smoker: HR = 1.33, 95% CI: 1.09, 1.62; previously married vs. currently married: HR = 1.55, 95% CI: 1.33, 1.79; and living alone vs. living with a partner: HR = 1.55, 95% CI: 1.35, 1.77) than for women (HR = 0.96, 95% CI: 0.84, 1.09; HR = 1.18, 95% CI: 1.05, 1.33; and HR = 1.21, 95% CI: 1.08, 1.35; respectively).

**Table 2:** Bivariate associations between the explanatory factors and mortality for men and women

	Men		Women		Testing for a gender difference in the bivariate associations (*p* value)^c^
	HR^a^	(95% CI)^b^	HR^a^	(95% CI)^b^	
Material factors					
Financial difficulties					
No	1	Ref.	1	Ref.	
Some	1.16	(1.02, 1.32)	1.28	(1.13, 1.46)	0.278
Big	1.41	(1.06, 1.87)	1.45	(1.13, 1.88)	0.977
Housing tenure					
Owned home	1	Ref.	1	Ref.	
Rented home	1.28	(1.15, 1.43)	1.29	(1.16, 1.44)	0.711
Health insurance					
Private	1	Ref.	1	Ref.	
Public	1.34	(1.18, 1.51)	1.25	(1.11, 1.40)	0.358
Employment-related factors					
Employment					
Employed	1	Ref.	1	Ref.	
Unemployed	1.84	(1.58, 2.14)	1.26	(0.97, 1.64)	0.012
Retired	1.10	(0.95, 1.26)	0.90	(0.71, 1.12)	0.313
Other	0.92	(0.47, 1.81)	0.86	(0.71, 1.05)	0.979
Occupation of the breadwinner					
Professional	1	Ref.	1	Ref.	
White collar	1.07	(0.91, 1.25)	1.17	(0.99, 1.38)	0.384
Blue collar	1.03	(0.89, 1.19)	1.31	(1.13, 1.52)	0.026
Not in the workforce	1.27	(0.87, 1.85)	1.22	(1.04, 1.44)	0.936
Behavioural factors					
Alcohol consumption					
No	1.20	(1.04, 1.38)	1.14	(1.01, 1.28)	0.617
Light	1	Ref.	1	Ref.	
Moderate	1.12	(0.94, 1.32)	1.11	(0.93, 1.32)	0.967
Heavy	1.71	(1.48, 1.98)	1.60	(1.30, 1.96)	0.527
Body mass index (BMI)					
Underweight	1.58	(1.20, 2.07)	1.57	(1.26, 1.96)	0.943
Normal weight	1	Ref.	1	Ref.	
Overweight	1.04	(0.94, 1.16)	1.04	(0.92, 1.18)	0.948
Obesity	1.36	(1.07, 1.74)	1.32	(1.11, 1.58)	0.792
Smoking					
Current	2.49	(2.05, 3.02)	2.06	(1.83, 2.33)	0.053
Former	1.33	(1.09, 1.62)	0.96	(0.84, 1.09)	0.008
Never	1	Ref.	1	Ref.	
Leisure activity					
Inactive	1.43	(1.21, 1.69)	1.50	(1.29, 1.75)	0.607
Little	1.28	(1.10, 1.50)	1.15	(0.99, 1.33)	0.410
Moderate	1.04	(0.92, 1.17)	1.03	(0.90, 1.17)	0.930
Active	1	Ref.	1	Ref.	
Sports activity					
Inactive	1.26	(1.09, 1.46)	1.41	(1.18, 1.69)	0.317
Little	1.12	(0.89, 1.43)	1.15	(0.90, 1.47)	0.856
Moderate	0.96	(0.78, 1.18)	1.09	(0.88, 1.35)	0.377
Active	1	Ref.	1	Ref.	
Family-related factors					
Marital status					
Currently married	1	Ref.	1	Ref.	
Previously married	1.55	(1.33, 1.79)	1.18	(1.05, 1.33)	0.008
Never married	1.55	(1.27, 1.90)	1.31	(1.09, 1.58)	0.300
Living arrangement					
Living with partner	1	Ref.	1	Ref.	
Living alone	1.55	(1.35, 1.77)	1.21	(1.08, 1.35)	0.008
Number of children					
0	1.10	(0.94, 1.28)	1.23	(1.04, 1.44)	0.288
1	1.01	(0.85, 1.19)	1.20	(1.01, 1.43)	0.128
2	1	Ref.	1	Ref.	
3 or more	0.97	(0.86, 1.09)	0.92	(0.81, 1.04)	0.663

*Notes*. ^a^*HR* Hazard ratios. ^b^*CI* Confidence interval. Mortality hazard ratios of the explanatory variable when controlling for education only. As the Cox regression models included age as timescale, it was unnecessary to also include age as a covariate in the models. ^c^A possible differential association of these explanatory factors with mortality for men and women was determined by adding an interaction term between the explanatory factor and gender and testing its statistical significance (*p* value)

### Contribution to educational inequalities in all-cause mortality

A statistically significant elevated mortality risk for the lowest educated men and women (HR = 1.58, 95% CI: 1.37, 1.83 for men; HR = 1.59, 95% CI: 1.25, 2.02 for women; Table 3) was substantially attenuated after accounting for material factors (67% for men, 51% for women), and it was no longer statistically significantly higher among men (HR = 1.18, 95% CI: 0.99, 1.40 for men; HR = 1.29, 95% CI: 1.00, 1.66 for women). Type of health insurance seemed to explain more of the excess mortality risk of the lowest educated men (53%) than of the lowest educated women (32%). Both employment-related factors together explained a similar share of the educational inequalities for both men (between 21 and 28%) and women (between 11 and 34%), but strong differences were found for the separate employment-related factors. Type of employment (and specifically unemployment) explained more of the educational inequalities for men (29%) than for women (− 7%), whereas occupation of the breadwinner seemed to explain more for women (41%) than for men (7%). Behavioural factors explained a similar proportion of the educational inequalities for men (between 11 and 36%) and women (between 13 and 37%). Family factors did not explain educational inequalities for either men or women; they even seemed to slightly strengthen the inequalities for both. When all risk factors were considered, a substantial part of the educational inequalities in mortality was explained for both men (between 33 and 62%) and women (between 28 and 71%). The increased HR of the lowest educated men compared to the highest educated men remained borderline statistically significant (HR = 1.22, 95% CI:1.00, 1.49). Results for low and mid educated men and women are presented in Additional file 1: Tables S2 and S3.

**Table 3:** Contributions of the explanatory factors to educational inequalities in all-cause mortality for lowest educated men and women

Models	Men						Women					
	Level of education			Change in educational inequality			Level of education			Change in educational inequality		
	Lowest		High	Absolute decline ^c^	Percentage decline ^c^		Lowest		High	Absolute decline^c^	Percentage decline^c^	
	HR^a^	(95% CI)^b^	Ref.		%	(95% CI)^d^	HR^a^	(95% CI)^b^	Ref.		%	(95% CI)^d^
0. No additional controls	1.58	(1.37, 1.83)	1				1.59	(1.25, 2.02)	1			
1. Material	1.18	(0.99, 1.40)	1	0.40	67%	(46%, 103%)	1.29	(1.00, 1.66)	1	0.30	51%	(29%, 93%)
Financial difficulties	1.51	(1.30, 1.75)	1	0.07	12%	(4%, 24%)	1.49	(1.17, 1.90)	1	0.10	17%	(9%, 34%)
Housing tenure	1.40	(1.20, 1.63)	1	0.18	31%	(18%, 49%)	1.41	(1.10, 1.81)	1	0.18	31%	(15%, 56%)
Health insurance	1.27	(1.07, 1.51)	1	0.31	53%	(31%, 81%)	1.40	(1.09, 1.80)	1	0.19	32%	(15%, 61%)
2. Employment-related	1.42	(1.18, 1.71)	1	0.16	28%	(3%, 58%)	1.39	(1.07, 1.81)	1	0.20	34%	(2%, 55%)
Employment	1.41	(1.21, 1.63)	1	0.17	29%	(20%, 45%)	1.63	(1.27, 2.08)	1	−0.04	−7%	(−22%, 2%)
Occ. of the breadwinner^e^	1.54	(1.28, 1.85)	1	0.04	7%	(−20%, 37%)	1.35	(1.04, 1.75)	1	0.24	41%	(8%, 59%)
3. Behavioural factors	1.39	(1.20, 1.62)	1	0.19	33%	(16%, 50%)	1.37	(1.06, 1.75)	1	0.22	37%	(15%, 77%)
Alcohol consumption	1.55	(1.34, 1.79)	1	0.03	5%	(−3, 14%)	1.61	(1.26, 2.06)	1	−0.02	−3%	(−20%, 10%)
BMI	1.55	(1.34, 1.79)	1	0.03	5%	(1%, 11%)	1.57	(1.23, 2.00)	1	0.02	3%	(−5%, 12%)
Smoking	1.50	(1.30, 1.73)	1	0.08	14%	(3%, 26%)	1.48	(1.16, 1.89)	1	0.11	19%	(4%, 41%)
Leisure activity	1.59	(1.38, 1.84)	1	−0.01	−2%	(−7%, 4%)	1.53	(1.20, 1.95)	1	0.06	10%	(1%, 22%)
Sports activity	1.49	(1.29, 1.72)	1	0.09	16%	(9%, 27%)	1.49	(1.17, 1.90)	1	0.10	17%	(8%, 34%)
4. Family-related factors	1.57	(1.36, 1.81)	1	0.01	2%	(−7%, 11%)	1.62	(1.27, 2.07)	1	−0.03	−5%	(−25%, 6%)
Marital status	1.57	(1.36, 1.81)	1	0.01	2%	(−4%, 10%)	1.61	(1.26, 2.06)	1	−0.02	−3%	(−22%, 6%)
Living arrangement	1.56	(1.35, 1.81)	1	0.02	3%	(−3%, 9%)	1.58	(1.24, 2.01)	1	0.01	2%	(−3%, 6%)
Number of children	1.56	(1.35, 1.81)	1	0.02	3%	(−2%, 10%)	1.65	(1.29, 2.10)	1	−0.06	−10%	(−26%, −1%)
5. All factors	1.22	(1.00, 1.49)	1	0.36	62%	(30%, 101%)	1.17	(0.89, 1.54)	1	0.42	71%	(28%, 123%)

*Notes*. ^a^*HR* Mortality hazard ratios. ^b^*CI* Confidence interval. ^c^Negative absolute and percentage declines indicate an increase in the educational inequality. ^d^Confidence intervals (CIs) of the percentage decline were calculated using bootstraps with 5000 repetitions; 1000 repetitions per imputed dataset. ^e^Occ.: Occupation

### Explaining educational inequalities in cause-specific mortality

All categories of explanatory variables seemed to explain more of the educational inequalities observed in cardiovascular mortality for men (88%, Table 4) than for women (12%). For women, educational inequalities in mortality from cardiovascular disease (HR = 2.08, 95% CI: 1.26, 3.44) were stronger than those in all-cause mortality (HR = 1.59, 95% CI: 1.25, 2.02), and they persisted even after controlling for all explanatory factors (HR = 1.95, 95% CI: 1.11, 3.41). For men, the risk factors explained less of the educational inequalities in cancer mortality (28%) than of the educational inequalities in all-cause mortality (62%). All explanatory variables, with the exception of family-related factors, appeared to explain more of the educational inequalities in cancer mortality for women (112%) than for men (28%). With regards to mortality from other diseases, material and family-related factors seemed to explain a larger part of the educational inequalities for men (94% and 10% respectively) than for women (58% and − 12% respectively), whereas employment-related and behavioural factors seemed to explain more for women (30% and 63% respectively) than for men (13% and 58% respectively). Our explanatory variables explained some of the elevated mortality risk from external causes for lowest educated women with respect to higher educated women (50%), but they did not contribute to an explanation for men.

**Table 4:** Contributions of the explanatory factors to educational inequalities in cause-specific mortality for lowest educated men and women

Models	Men					Women				
	Level of education			Change in educational inequality		Level of education			Change in educational inequality	
	Lowest		High	Absolute decline^c^	Percentage decline^c^	Lowest		High	Absolute decline^c^	Percentage decline^c^
	HR^a^	(95% CI)^b^	Ref.			HR^a^	(95% CI)^b^	Ref.		
Mortality from cardiovascular disease^d^										
0. No additional controls	1.58	(1.23, 2.04)	1			2.08	(1.26, 3.44)	1		
1. Material	1.25	(0.91, 1.71)	1	0.33	57%	2.13	(1.27, 3.57)	1	−0.05	−5%
2. Employment-related	1.17	(0.84, 1.62)	1	0.41	71%	1.90	(1.11, 3.24)	1	0.18	17%
3. Behavioural	1.39	(1.06, 1.81)	1	0.19	33%	1.87	(1.11, 3.16)	1	0.21	19%
4. Family-related	1.57	(1.21, 2.03)	1	0.01	2%	2.02	(1.22, 3.32)	1	0.06	6%
5. All factors	1.07	(0.75, 1.52)	1	0.51	88%	1.95	(1.11, 3.41)	1	0.13	12%
Cancer mortality^e^										
0. No additional controls	1.50	(1.20, 1.89)	1			1.33	(0.91, 1.95)	1		
1. Material	1.26	(0.96, 1.66)	1	0.24	48%	1.04	(0.70, 1.55)	1	0.29	88%
2. Employment-related	1.52	(1.14, 2.04)	1	−0.02	−4%	1.14	(0.75, 1.73)	1	0.19	58%
3. Behavioural	1.33	(1.05, 1.69)	1	0.17	34%	1.21	(0.82, 1.79)	1	0.12	36%
4. Family-related	1.50	(1.19, 1.88)	1	0.00	0%	1.38	(0.94, 2.04)	1	−0.05	−15%
5. All factors	1.36	(1.00, 1.86)	1	0.14	28%	0.96	(0.61, 1.49)	1	0.37	112%
Mortality from other diseases^f^										
0. No additional controls	1.31	(0.99, 1.74)	1			1.43	(0.94, 2.19)	1		
1. Material	1.02	(0.73, 1.43)	1	0.29	94%	1.18	(0.77, 1.82)	1	0.25	58%
2. Employment-related	1.27	(0.89, 1.81)	1	0.04	13%	1.30	(0.83, 2.05)	1	0.13	30%
3. Behavioural	1.13	(0.83, 1.53)	1	0.18	58%	1.16	(0.75, 1.80)	1	0.27	63%
4. Family-related	1.28	(0.96, 1.71)	1	0.03	10%	1.48	(0.96, 2.27)	1	−0.05	−12%
5. All factors	1.03	(0.70, 1.51)	1	0.28	90%	1.06	(0.66, 1.72)	1	0.37	86%
Mortality from external causes^g^										
0. No additional controls	0.95	(0.39, 2.32)	1			2.05	(0.48, 8.77)	1		
1. Material	1.17	(0.38, 3.63)	1	−0.22	− 440%	1.66	(0.35, 8.00)	1	0.39	37%
2. Employment-related	1.21	(0.32, 4.54)	1	−0.26	− 520%	1.27	(0.30, 5.45)	1	0.78	74%
3. Behavioural	1.04	(0.41, 2.61)	1	−0.09	−180%	2.27	(0.51, 10.04)	1	−0.22	−21%
4. Family-related	0.98	(0.39, 2.44)	1	−0.03	−60%	2.20	(0.50, 9.65)	1	−0.15	−14%
5. All factors	1.42	(0.37, 5.42)	1	−0.47	− 940%	1.52	(0.28, 8.22)	1	0.53	50%

*Notes*. ^a^*HR* Mortality hazard ratios. ^b^*CI* Confidence interval. ^c^Negative absolute and percentage declines indicate an increase in the educational inequality. ^d^Mortality from cardiovascular disease includes deaths with International Classification of Diseases (ICD) [36] codes between I00 and I99. ^e^Cancer mortality includes deaths with ICD codes between C00 and D48. ^f^Mortality from other diseases includes deaths with all other ICD codes than those included in cardiovascular disease, cancer or external causes. ^g^Mortality from external causes includes deaths with ICD codes between V01 and Y89

## Discussion

Educational gradients in mortality were found for both men and women. Although a substantial and reasonably similar part of the educational inequalities in mortality was explained by all material, employment-related, behavioural and family-related factors together for both men (62%) and women (71%), the specific contributions of some factors differed between men and women. Specifically, type of employment explained more of the educational inequalities in all-cause mortality for men than for women, whereas the occupational class of the breadwinner explained more for women than for men. Our results also suggested that material and employment-related factors contribute more to inequalities in mortality from cardiovascular diseases for men than for women, but they explained more of the inequalities in cancer mortality for women than for men.

### Methodological considerations

Besides a mortality follow-up of more than 20 years, a major strength of this study is the inclusion of a broad selection of material, employment-related, behavioural and family-related factors. However, these factors were self-reported and may contain measurement error. If biases in self-reports differed by education or gender, for which evidence exists, this may have affected our results. For example, in the French GAZEL study, men underestimated their weight and overestimated their height less than women, and high educated men and women overestimated their height less than their low educated counterparts [41]. Differences in self-reporting biases by gender or education have also been found for other (behavioural) factors, including physical activity [42] and smoking [43]. Yet, the exact direction of self-report misclassification by gender and socioeconomic status is less clear.

In this study, we examined the contribution of single measurements of material, employment, behavioural and family factors to educational inequalities in mortality. It may be argued that using multiple measurements of the explanatory variables would be better as it allows to account for possible changes over time in inequalities in these variables. Recent studies have shown that the contribution of behavioural factors to socioeconomic inequalities in mortality was (slightly) larger when multiple measurements over time of these factors were included [44–46]. Using the same GLOBE data, Oude Groeniger and colleagues also found a larger contribution of behavioural factors to educational inequalities in mortality when multiple measurements were used, but a smaller contribution of material factors [47]. Yet, another study found a slightly higher attenuation of educational inequalities in mortality for men when behavioural, psychosocial, biomedical risk factors and employment were measured twice (63%, compared to 53% when only the baseline measurement was used), but no change in the attenuation for women [48]. We believe our overall conclusions to be valid, as by using only a single measurement we may have slightly underestimated the contribution of behavioural factors and slightly overestimated the contribution of material factors to socioeconomic inequalities in mortality.

Longitudinal data on the explanatory variables was available but only for two subsamples of the GLOBE study (*N* = 5667). After exclusion of the chronically ill and respondents with missing information on any of the other variables, the final sample would be even smaller. As our main focus was on estimating gender differences in the explanations of educational inequalities in mortality, we decided to use the baseline sample. We believe that misclassification bias due to changes in educational status during the 23 years of follow-up is relatively small as our sample exists of individuals aged 25 years and over, who are likely to have finished their education. Second, using the longitudinal data from the GLOBE study, Oude Groeniger and colleagues found that although the contribution of behavioural and material factors to explaining educational inequalities in mortality changed when these factors were measured multiple times, they did not observe clear gender differences in these changes [47]. Based on these findings, we therefore do not believe that differential changes in the explanatory factors by gender would contribute to explaining the observed differences.

Despite the fact that we used a broad set of material, employment-related, behavioural and family-related factors in our study, it may be argued that inclusion of more specific explanatory factors should be considered. To the extent that the prevalence of such specific measures and their association with mortality differs between men and women, including them could have led to more specific estimations of the contribution of the explanatory factors. For example, dietary intake, e.g. fruit and vegetable consumption, is educationally patterned [49], and may play a more important role in explaining educational inequalities in mortality than a summary measure such as BMI. Unfortunately our data did not allow us to include these more specific measures, but we strongly encourage future studies to consider these factors and examine their contribution to explaining educational inequalities in mortality.

Finally, although the study population of the GLOBE study is reasonably representative for the Dutch ethnic population, residents of non-Western ethnicities were almost absent in the baseline measurement [31]. Generalizing our findings to other populations or countries should therefore be done with caution.

### Interpretation

Overall, we found a similar contribution of material and behavioural factors to socioeconomic inequalities in all-cause mortality for both men and women. Of all explanatory variables, material factors contributed most to the explanation of educational inequalities in mortality. This finding is in line with previous research [7, 8, 10]. Behavioural factors also provided a substantial contribution to educational inequalities in all-cause mortality in our study; our estimates fit the broad range of contributions reported by previous studies, including a 56% reduction of the educational gradient in mortality in the British Whitehall study and a 17% reduction in the French GAZEL study [12].

However, type of employment was more important in explaining educational inequalities in all-cause mortality for men than for women. Unemployment was more strongly associated with mortality and more strongly educational patterned among men than among women. In contrast, the occupational class of the breadwinner was more important in explaining educational inequalities in mortality for women than for men. Although a weaker educational gradient in blue-collar occupation of the breadwinner was found among women than among men, its stronger association with mortality for women resulted in a larger contribution of this factor to the explanation of educational inequalities in mortality for women than for men. As a large proportion of the women in our sample were outside the labour force, i.e. 57% of all women, the occupational class of the breadwinner may thus possibly be a better representative of their social class than their own employment status. However, for men it seemed that having a job outweighed the prestige associated with that job. This is likely to explain the gender differences in the contribution of our employment-related factors to explaining educational inequalities in mortality. However, we did not find evidence of gender differences in the explanation of educational inequalities in mortality for the other explanatory variables. Thus even though there were gender differences in the educational gradient as well as the association with mortality for some of our other variables (e.g. being previously married and living alone), our results showed that these differences were not large enough to lead to gender differences in explanations or were compensating each other.

Besides material, employment-related and behavioural factors, we also examined whether family-related factors play a role in explaining educational inequalities in mortality, as we know that they are associated with mortality. Our results suggest that marital status, living arrangement and number of children did not contribute to the explanation of socioeconomic inequalities in mortality for either men or women. For example, even though being previously married was more strongly associated with mortality for men than for women, marital status was less socially patterned among men than women, and thus no significant differences in the contribution of marital status to socioeconomic inequalities in mortality by gender were found. Our results may well have depended on the relatively broad measures of family-related factors, such as legal marital status and the number of children in the household, that were included. Therefore, we recommend future studies to take into account more specific family-related factors or even full family life histories in a longitudinal analysis, to advance our knowledge on how family-related factors may contribute to educational inequalities in mortality.

In the cause-specific analysis, we found that our explanatory variables explained a substantial part of educational inequalities in mortality for cardiovascular disease (CVD) for men, but not for women. Our results are partly in line with previous results [50]; a substantial contribution of material factors in the explanation of educational inequalities in CVD mortality for men, and the largest contribution of behavioural factors in the explanation of educational inequalities in CVD mortality for women. Surprisingly however, material factors did not contribute to the explanation of educational inequalities in CVD mortality for women, but it explained a substantial part of their educational inequalities in all-cause mortality and mortality from the other causes (cancer, other diseases, and external causes). This warrants further investigation.

## Conclusions

Our findings highlight the importance of a gender perspective in research on educational inequalities in mortality and the factors contributing to the explanation of these inequalities, as the contributions of these factors differed for men and women. Policies targeting the reduction of educational inequalities in mortality should focus on improving material circumstances and discouraging unhealthy behaviours, and would also benefit from a gendered approach as interventions addressing specific factors may have differential effects on educational inequalities for men and women.

In conclusion, unemployment seemed more important in explaining educational inequalities in mortality for men than for women, whereas social class of the breadwinner was more important for the explanation for women than for men. A full understanding of educational inequalities in mortality thus benefits from a gender perspective, particularly when considering employment-related factors.

## Additional file

Additional file 1: **Table S1.** Age-standardised distribution of educational level for men and women, at baseline 1991. **Table S2.** Contributions of the explanatory factors to educational inequalities in all-cause mortality for low educated men and women. **Table S3.** Contributions of the explanatory factors to educational inequalities in all-cause mortality for mid educated men and women. (DOCX 63 kb)

## Abbreviations

BMI: Body Mass Index
CI: Confidence Interval
CVD: Cardiovascular Disease
HR: Hazard Ratio
ICD: International Classification of Diseases
ISCED: International Standard Classification of Education
