# Supplementary material for: What’s the difference? A gender perspective on understanding educational inequalities in all-cause and cause-specific mortality
Source: BMC Public Health. 2018 Sep 10;18:1105. doi: 10.1186/s12889-018-5940-5 (PMC6131918; doi:10.1186/s12889-018-5940-5)
Supplement: Supplementary file 1 — Table S1. Age-standardised distribution of educational level for men and women, at baseline 1991. Table S2. Contributions of the explanatory factors to educational inequalities in all-cause mortality for low educated men and women. Table S3. Contributions of the explanatory factors to educational inequalities in all-cause mortality for mid educated men and women. (DOCX 63 kb) [file 12889_2018_5940_MOESM1_ESM.docx]

**Additional file**

**Table S1 ─ Age-standardised distribution of educational level for men and women, at baseline 1991**

|  | **Men** | | **Women** | |
| --- | --- | --- | --- | --- |
| **Education** | **Proportion** | **(95% CI) ^a^** | **Proportion** | **(95% CI) ^a^** |
| Lowest | 19.1 | (18.1, 20.2) | 25.2 | (24.2, 26.2) |
| Low | 33.2 | (31.9, 34.4) | 47.3 | (46.2, 48.5) |
| Mid | 22.2 | (21.2, 23.3) | 17.0 | (16.2, 17.9) |
| High | 25.5 | (24.3, 26.6) | 10.4 | (9.7, 11.1) |

*Notes*. ^a^ CI: Confidence interval

**Table S2 ─ Contributions of the explanatory factors to educational inequalities in all-cause mortality for low educated men and women**

|  | | **Men** | | | | | | | **Women** | | | | | |
| --- | --- | --- | --- | --- | --- | --- | --- | --- | --- | --- | --- | --- | --- | --- |
|  | | **Level of education** | | | | **Change in  educational inequality** | | | **Level of education** | | | **Change in  educational inequality** | | |
|  | | Low | | | High | Absolute  decline ^c^ | Percentage  decline ^c^ | | Low | | High | Absolute  decline ^c^ | Percentage  decline ^c^ | |
| **Models** | | HR ^a^ | (95% CI) ^b^ | | Ref. |  | % | (95% CI) ^d^ | HR ^a^ | (95% CI) ^b^ | Ref. |  | % | (95% CI) ^d^ |
| **0. No additional controls** | | **1.28** | | **(1.11, 1.47)** | **1** |  |  |  | **1.44** | **(1.13, 1.83)** | **1** |  |  |  |
| **1. Material** | | **1.03** | | **(0.88, 1.21)** | **1** | **0.25** | **89%** | **(51%, 202%)** | **1.27** | **(0.99, 1.63)** | **1** | **0.17** | **39%** | **(19%, 93%)** |
|  | Financial difficulties | 1.24 | | (1.08, 1.43) | 1 | 0.04 | 14% | (3%, 31%) | 1.40 | (1.10, 1.78) | 1 | 0.04 | 9% | (4%, 26%) |
|  | Housing tenure | 1.16 | | (1.00, 1.34) | 1 | 0.12 | 43% | (22%, 97%) | 1.34 | (1.05, 1.71) | 1 | 0.10 | 23% | (10%, 53%) |
|  | Health insurance | 1.09 | | (0.94, 1.28) | 1 | 0.19 | 68% | (34%, 152%) | 1.32 | (1.04, 1.69) | 1 | 0.12 | 27% | (12%, 64%) |
| **2. Employment-related** | | **1.22** | | **(1.03, 1.44)** | **1** | **0.06** | **21%** | **(-15%, 77%)** | **1.31** | **(1.02, 1.69)** | **1** | **0.13** | **30%** | **(-3%, 51%)** |
|  | Employment | 1.22 | | (1.06, 1.40) | 1 | 0.06 | 21% | (12%, 53%) | 1.47 | (1.15, 1.88) | 1 | -0.03 | -7% | (-30%, 2%) |
|  | Occ. of the breadwinner ^e^ | 1.24 | | (1.05, 1.47) | 1 | 0.04 | 14% | (-27%, 60%) | 1.28 | (0.99, 1.64) | 1 | 0.16 | 36% | (6%, 63%) |
| **3. Behavioural factors** | | **1.18** | | **(1.02, 1.36)** | **1** | **0.10** | **36%** | **(10%, 75%)** | **1.33** | **(1.04, 1.69)** | **1** | **0.11** | **25%** | **(3%, 78%)** |
|  | Alcohol consumption | 1.26 | | (1.10, 1.45) | 1 | 0.02 | 7% | (-7%, 17%) | 1.47 | (1.15, 1.87) | 1 | -0.03 | -7% | (-26%, 6%) |
|  | BMI | 1.25 | | (1.09, 1.44) | 1 | 0.03 | 11% | (0%, 24%) | 1.44 | (1.13, 1.84) | 1 | 0.00 | 0% | (-10%, 7%) |
|  | Smoking | 1.26 | | (1.09, 1.45) | 1 | 0.02 | 7% | (-12%, 29%) | 1.38 | (1.08, 1.75) | 1 | 0.06 | 14% | (-2%, 48%) |
|  | Leisure activity | 1.27 | | (1.10, 1.46) | 1 | 0.01 | 4% | (-4%, 12%) | 1.41 | (1.11, 1.79) | 1 | 0.03 | 7% | (-2%, 21%) |
|  | Sports activity | 1.23 | | (1.06, 1.41) | 1 | 0.05 | 18% | (9%, 45%) | 1.39 | (1.09, 1.77) | 1 | 0.05 | 11% | (2%, 33%) |
| **4. Family-related factors** | | **1.30** | | **(1.13, 1.50)** | **1** | **-0.02** | **-7%** | **(-27%, 3%)** | **1.50** | **(1.17, 1.91)** | **1** | **-0.06** | **-14%** | **(-46%, -0%)** |
|  | Marital status | 1.31 | | (1.14, 1.51) | 1 | -0.03 | -11% | (-28%, -1%) | 1.49 | (1.17, 1.90) | 1 | -0.05 | -11% | (-41%, 0%) |
|  | Living arrangement | 1.30 | | (1.13, 1.49) | 1 | -0.02 | -7% | (-21%, 1%) | 1.46 | (1.15, 1.85) | 1 | -0.02 | -5% | (-16%, 0%) |
|  | Number of children | 1.27 | | (1.11, 1.47) | 1 | 0.01 | 4% | (-4%, 11%) | 1.50 | (1.17, 1.91) | 1 | -0.06 | -14% | (-40%, -2%) |
| **5. All factors** | | **1.11** | | **(0.93, 1.33)** | **1** | **0.17** | **61%** | **(12%, 152%)** | **1.21** | **(0.93, 1.58)** | **1** | **0.23** | **52%** | **(5%, 120%)** |

*Notes*. ^a^ HR: Mortality hazard ratios. ^b^ CI: Confidence interval. ^c^ Negative absolute and percentage declines indicate an increase in the educational inequality. ^d^ Confidence intervals (CIs) of the percentage decline were calculated using bootstraps with 5000 repetitions; 1000 repetitions per imputed dataset. ^e^ Occ.: Occupation.

**Table S3 ─ Contributions of the explanatory factors to educational inequalities in all-cause mortality for mid educated men and women**

|  | | **Men** | | | | | | **Women** | | | | | |
| --- | --- | --- | --- | --- | --- | --- | --- | --- | --- | --- | --- | --- | --- |
|  | | **Level of education** | | | **Change in  educational inequality** | | | **Level of education** | | | **Change in  educational inequality** | | |
|  | | Mid | | High | Absolute  decline ^c^ | Percentage  decline ^c^ | | Mid | | High | Absolute  decline ^c^ | Percentage  decline ^c^ | |
| **Models** | | HR ^a^ | (95% CI) ^b^ | Ref. |  | % | (95% CI) ^d^ | HR ^a^ | (95% CI) ^b^ | Ref. |  | % | (95% CI) ^d^ |
| **0. No additional controls** | | **1.18** | **(1.01, 1.38)** | **1** |  |  |  | **1.46** | **(1.11, 1.91)** | **1** |  |  |  |
| **1. Material** | | **1.06** | **(0.90, 1.24)** | **1** | **0.12** | **67%** | **(27%, 378%)** | **1.37** | **(1.05, 1.80)** | **1** | **0.09** | **20%** | **(8%, 55%)** |
|  | Financial difficulties | 1.17 | (1.00, 1.37) | 1 | 0.01 | 6% | (0%, 42%) | 1.44 | (1.10, 1.89) | 1 | 0.02 | 4% | (-1%, 13%) |
|  | Housing tenure | 1.13 | (0.96, 1.32) | 1 | 0.05 | 28% | (11%, 164%) | 1.41 | (1.07, 1.85) | 1 | 0.05 | 11% | (4%, 33%) |
|  | Health insurance | 1.09 | (0.93, 1.28) | 1 | 0.09 | 50% | (18%, 267%) | 1.40 | (1.07, 1.84) | 1 | 0.06 | 13% | (5%, 36%) |
| **2. Employment-related** | | **1.14** | **(0.97, 1.35)** | **1** | **0.04** | **22%** | **(-20%, 115%)** | **1.41** | **(1.07, 1.85)** | **1** | **0.05** | **11%** | **(-8%, 21%)** |
|  | Employment | 1.15 | (0.98, 1.34) | 1 | 0.03 | 17% | (2%, 106%) | 1.49 | (1.14, 1.95) | 1 | -0.03 | -7% | (-26%, -0%) |
|  | Occ. of the breadwinner ^e^ | 1.16 | (0.98, 1.37) | 1 | 0.02 | 11% | (-37%, 67%) | 1.37 | (1.05, 1.81) | 1 | 0.09 | 20% | (3%, 35%) |
| **3. Behavioural factors** | | **1.16** | **(0.99, 1.35)** | **1** | **0.02** | **11%** | **(-38%, 103%)** | **1.40** | **(1.06, 1.83)** | **1** | **0.06** | **13%** | **(-12%, 58%)** |
|  | Alcohol consumption | 1.17 | (1.00, 1.37) | 1 | 0.01 | 6% | (-25%, 33%) | 1.47 | (1.12, 1.92) | 1 | -0.01 | -2% | (-15%, 14%) |
|  | BMI | 1.17 | (1.00, 1.37) | 1 | 0.01 | 6% | (-4%, 36%) | 1.48 | (1.13, 1.94) | 1 | -0.02 | -4% | (-17%, 2%) |
|  | Smoking | 1.19 | (1.02, 1.40) | 1 | -0.01 | -6% | (-72%, 37%) | 1.40 | (1.07, 1.84) | 1 | 0.06 | 13% | (-7%, 48%) |
|  | Leisure activity | 1.18 | (1.01, 1.38) | 1 | 0.00 | 0% | (-18%, 21%) | 1.44 | (1.10, 1.88) | 1 | 0.02 | 4% | (-4%, 22%) |
|  | Sports activity | 1.16 | (0.99, 1.36) | 1 | 0.02 | 11% | (-3%, 62%) | 1.45 | (1.11, 1.90) | 1 | 0.01 | 2% | (-8%, 16%) |
| **4. Family-related factors** | | **1.22** | **(1.04, 1.42)** | **1** | **-0.04** | **-22%** | **(-117%, 3%)** | **1.49** | **(1.13, 1.95)** | **1** | **-0.03** | **-7%** | **(-27%, 5%)** |
|  | Marital status | 1.22 | (1.04, 1.42) | 1 | -0.04 | -22% | (-109%, -2%) | 1.48 | (1.13, 1.94) | 1 | -0.02 | -4% | (-21%, 4%) |
|  | Living arrangement | 1.21 | (1.03, 1.41) | 1 | -0.03 | -17% | (-78%, 2%) | 1.46 | (1.12, 1.92) | 1 | 0.00 | 0% | (-9%, 6%) |
|  | Number of children | 1.17 | (1.00, 1.37) | 1 | 0.01 | 6% | (-8%, 34%) | 1.49 | (1.14, 1.96) | 1 | -0.03 | -7% | (-24%, 2%) |
| **5. All factors** | | **1.12** | **(0.95, 1.32)** | **1** | **0.06** | **33%** | **(-45%, 201%)** | **1.33** | **(1.01, 1.76)** | **1** | **0.13** | **28%** | **(-15%, 72%)** |

*Notes*. ^a^ HR: Mortality hazard ratios. ^b^ CI: Confidence interval. ^c^ Negative absolute and percentage declines indicate an increase in the educational inequality. ^d^ Confidence intervals (CIs) of the percentage decline were calculated using bootstraps with 5000 repetitions; 1000 repetitions per imputed dataset. ^e^ Occ.: Occupation.
